# Gut microbiota alterations and systemic inflammation in community-acquired pneumonia: a prospective gut-lung axis study

**DOI:** 10.3389/fimmu.2025.1715214

**Published:** 2025-11-25

**Authors:** Xue-Qin Yang, Qian Tang, Yuan-Jun Xiong, Yang Zhao, Xiao-Hong Yin, Zhe Xu

**Affiliations:** Department of Pharmacy, Guangyuan Central Hospital, Affiliated Hospital of North Sichuan Medical College, Guangyuan, Sichuan, China

**Keywords:** gut-lung axis, community-acquired pneumonia, gut microbiota, lipopolysaccharide, inflammation

## Abstract

Up to now, only a few scattered studies have provided some evidence for the relationship between gut microbiota and community-acquired pneumonia (CAP), and the mechanisms by which gut microbiota contributes to the occurrence and development of CAP via the gut-lung axis require further investigation. In this study, fecal and serum samples from CAP patients and healthy controls were analyzed using 16S rRNA gene sequencing and enzyme-linked immunosorbent assay. The results showed that compared with healthy controls, alpha-diversity of gut microbiota in CAP patients was significantly reduced, and beta-diversity was significantly different at operational taxonomic units (OTUs), class, order, family, genus, and species levels. The abundance of short-chain fatty acid-producing genera in CAP patients decreased significantly, such as Blautia and Agathobacter. Meanwhile genera including Gemmiger, Enterocloster, and Thomasclavelia were enriched in the CAP. Functional predictions based on KEGG Orthologies suggested that the gut microbiota of CAP patients was enriched in pathways related to carbohydrate metabolism and bacterial infection. Serum detection revealed that the levels of lipopolysaccharide (LPS), TNF-α, and IL-6 were significantly increased in CAP patients. Our findings suggest that gut microbiota dysbiosis in CAP patients is associated with increased translocation of LPS into the bloodstream and activation of systemic inflammation, indicating that the gut-lung axis may play a potential role in the pathogenesis of CAP.

## 1 Introduction

Community-acquired pneumonia (CAP) is an infectious pulmonary inflammation acquired outside of hospitals. It has a substantial global impact and stands as the leading cause of infection-related deaths worldwide (1, 2). According to a report by the World Health Organization (WHO), CAP causes 4 million deaths every year, accounting for 7% of the total annual deaths in the world (3). Even in the post-COVID-19 era, CAP remains a core issue in the field of global public health.

Previous studies have confirmed the bidirectional effect of the gut-lung axis: pulmonary diseases significantly affect the composition and function of the gut microbiota, while the gut microbiota also plays an indispensable role in the host’s defense against respiratory infections through immunomodulatory mechanisms (4, 5). The dysbiosis of gut microbiota can promote the migration of pathogenic bacteria to the lungs, and directly or indirectly regulate pulmonary immune responses through its metabolites, thus inducing or aggravating lung injury (6). Emerging evidence suggests that microbial therapy may serve as a novel approach for the prevention and treatment of various respiratory diseases, such as chronic obstructive pulmonary disease (COPD), lung cancer, and asthma (7). Up to now, only a few scattered studies have shown that when CAP patients develop pulmonary infections, there is a high probability of crosstalk between the gut-lung axis, along with a reduction in secondary bile acids and excessive proliferation of pro-inflammatory bacteria in their intestines (8, 9). However, the role of the interaction between gut microbiota and inflammatory reaction in the occurrence and development of CAP, as well as its underlying regulatory mechanisms, remain to be further in-depth explored.

Gut microbiota dysbiosis can impair intestinal barrier integrity, leading to increased translocation of lipopolysaccharide (LPS)—a component of the cell wall of Gram-negative bacteria—into the bloodstream (10, 11). LPS can activate the Toll-like receptor 4 (TLR4)/nuclear factor kappaB (NF-κB) signaling pathway, and induce the release of pro-inflammatory cytokines, such as tumor necrosis factor-α (TNF-α) and interleukin-6 (IL-6), thus aggravating pulmonary inflammatory reaction (12). In this process, LPS, as a key medium, links gut microbiota dysbiosis to the activation of the inflammatory cascade (13). Therefore, comprehensive analysis of the gut microbiota and the LPS-mediated inflammatory cascade can provide a new perspective for revealing the pathogenesis of CAP.

In this study, 16S rRNA gene sequence analysis and serum inflammatory indicator analysis were performed on fecal and blood samples from CAP patients and healthy controls. The results showed that gut microbiota dysbiosis may promote the release of LPS in blood, increase the levels of high-sensitivity C-reactive protein, TNF-α and IL-6, and trigger a metabolic inflammatory cascade.

## 2 Materials and methods

### 2.1 Participants

This study was conducted in accordance with the Declaration of Helsinki, and approved by the Ethics Committee of Guangyuan Central Hospital (Approval No.: GYZXLL202106, approval date: 2021-08-26). We prospectively recruited 31 inpatients with CAP, aged ≥ 18 years. The diagnosis of CAP was based on the official clinical practice guidelines of the American Thoracic Society (ATS) and the Infectious Diseases Society of America (IDSA) (14). Clinical data, including age, sex, smoking history, pathogen information, pneumonia severity index, complete blood count (CBC), and C-reactive protein (CRP) levels, were collected from the electronic medical records of the hospital. Patients exposed to antibiotics before hospitalization were excluded. In addition, 19 healthy adult volunteers without acute infections or underlying diseases were recruited as controls. Informed consent was obtained from all subjects involved in the study.

### 2.2 Sample collection

Fecal and blood samples were collected from healthy controls (HC group) and patients with CAP (CAP group) before medication administration. Fresh fecal samples were collected under sterile conditions: the outer surface was discarded, the inner material was divided into two equal parts, and then stored at -80 °C for microbiome analysis (8). Fasting blood samples were divided into three aliquots: two aliquots were used for routine blood tests and high-sensitivity C-reactive protein (hs-CRP) detection in the hospital, and the third aliquot was used for serum separation, followed by storage at -80 °C for enzyme-linked immunosorbent assay (ELISA) detection.

### 2.3 DNA extraction

Total community genomic DNA was extracted using the E.Z.N.A™ MagBind Soil DNA Kit (Omega, M5635-02, USA), and the concentration was measured with a Qubit 4.0 fluorometer (Thermo, USA) to ensure sufficient high-quality DNA was obtained.

### 2.4 16S rRNA gene amplification, library construction and sequencing

Targeting the V3-V4 hypervariable regions of the bacterial 16S rRNA gene, PCR amplification was performed immediately after DNA extraction. Hieff NGS™ DNA Selection Beads (Yeasen, 10105ES03, China) were used to purify free primers and primer dimers from the amplification products. The purified products were sent to Sangon Biotech (Shanghai) for library construction, and sequencing was completed using the Illumina MiSeq system (Illumina MiSeq, USA).

### 2.5 Functional prediction

Functional prediction analysis of bacteria was performed using PICRUSt2 (Version 2.5.2) software. By comparing the existing 16S rRNA gene sequencing data with the microbial reference genome database containing known metabolic functions, the prediction of bacterial metabolic functions was achieved.

### 2.6 Enzyme-linked immunosorbent assay

The concentrations of LPS, TNF-α, IL-6 in serum samples were quantitatively detected using ELISA kits (Jiangsu Meimian Industrial Co. Ltd., China). The catalog numbers of LPS, TNF-α, and IL-6 are MM-1309H1, MM-0122H2 and MM-0049H2 respectively. Among them, LPS was measured using a competitive kit with a limit of detection of 20 ng/L, a sensitivity of 7.5 ng/L, an inter-assay variation of 15%, and an intra-assay variation of 10%; serum samples were processed strictly in accordance with the kit instructions to ensure the accuracy of the detection results.

### 2.7 Statistical analysis

All statistical analyses were performed using R software (Version 4.3.2). Differences in the microbiota were identified using the Wilcoxon test, while other data were analyzed via the independent samples t-test, chi-square test, and Fisher’s exact test; Spearman’s rank correlation test was used for correlation analysis (15). Linear discriminant analysis effect size (LEfSe) was employed to screen for taxa significantly associated with sample groups (logarithmic LDA score threshold > 2.0). This method combines the nonparametric Kruskal-Wallis test for feature selection and uses linear discriminant analysis (LDA) to estimate the effect size of taxa with differential abundance. Categorical variables were expressed as absolute numbers and relative frequencies; continuous variables with a normal distribution were presented as mean ± standard deviation (SD), while those with a non-normal distribution were expressed as median and interquartile range (IQR) (16). Values of *p* < 0.05 were considered statistically significant.

## 3 Results

### 3.1 Clinical characteristics of CAP patients

In this study, a total of 31 CAP patients and 19 healthy controls were recruited. **Table 1** summarizes the age, sex, smoking history, complications, pathogenic bacteria, and pneumonia severity index of all participants. Pathogenic detection results were positive in 24 patients (77.4%). Among them, only bacterial infection (including Streptococcus pneumoniae, Haemophilus influenzae, Pseudomonas aeruginosa, etc.) accounted for the highest proportion (29.03%), followed by atypical pathogen infection (mainly mycoplasma pneumoniae, 16.1%), and fungal infection only accounted for 12.9%. One patient (3.2%) was diagnosed with co-infection of Mycoplasma pneumoniae and bacteria, and 5 patients (16.1%) were diagnosed with concurrent bacterial and fungal infections.

**Table 1:** Demographic data of enrolled patients and healthy controls.

Characteristic	CAP group	HC group	P value
Age (mean ± SD)	52.23 ± 14.47	50.53 ± 12.18	0.47
Sex			0.76
Male, n (%)	12 (38.7)	6 (31.6)	
Female, n (%)	19 (61.3)	13 (68.4)	
Smoke	8/31 (25.8%)	3/19 (15.8)	0.75
Complication			
Respiratory diseases	16/31 (51.6%)		
Type 2 diabetes mellitus	2/31 (6.5%)		
Hypertension	4/31 (12.9%)		
Coronary Artery Disease	8/31 (25.8%)		
Pathogens			
Atypical Pathogens Only, n (%)	5/31 (16.1%)		
Bacteria Only, n (%)	9/31 (29.03%)		
Fungi Only, n (%)	4/31 (12.9%)		
Atypical Pathogens + Bacteria, n (%)	1/31 (3.2%)		
Bacteria + Fungi, n (%)	5/31 (16.1%)		
Undetected, n (%)	7/31 (22.6%)		
Pneumonia Severity Index			
Class I-III, n (%)	30/31 (96.8%)		
Class IV, n (%)	1/31 (3.2%)		

The counts of white blood cells (WBC), neutrophils (Neut), and lymphocytes (Lym), as well as the levels of procalcitonin (PCT) and high-sensitivity C-reactive protein (hs-CRP), in patients at admission and healthy controls are all summarized in **Table 2**. The lymphocyte count of CAP group was significantly lower than that of HC group (*p* < 0.001), while the level of hs-CRP was significantly higher than that of HC group (*p* < 0.001). There were no significant differences in the other indexes between the two groups.

**Table 2:** Clinical indexes of patients and healthy controls at admission.

Indicator	CAP group	HC group	*P* value
WBC (109/L)	6.69 (4.91,7.96)	6.90 (6.49,7.35)	0.39
Neut (109/L)	4.81 (3.51,5.93)	3.97 (3.67,4.45)	0.16
Lym (109/L)	1.26 ± 0.50	2.13 ± 0.44	< 0.001
PCT (ng/ml)	0.13 (0.08, 0.21)	0.10 (0.06, 0.28)	0.49
Hs-CRP (mg/L)	9.96 (2.35, 31.26)	0.28 (0.17, 0.36)	< 0.001

### 3.2 Alpha- and beta-diversities of gut microbiota in CAP patients

**Figure 1:**
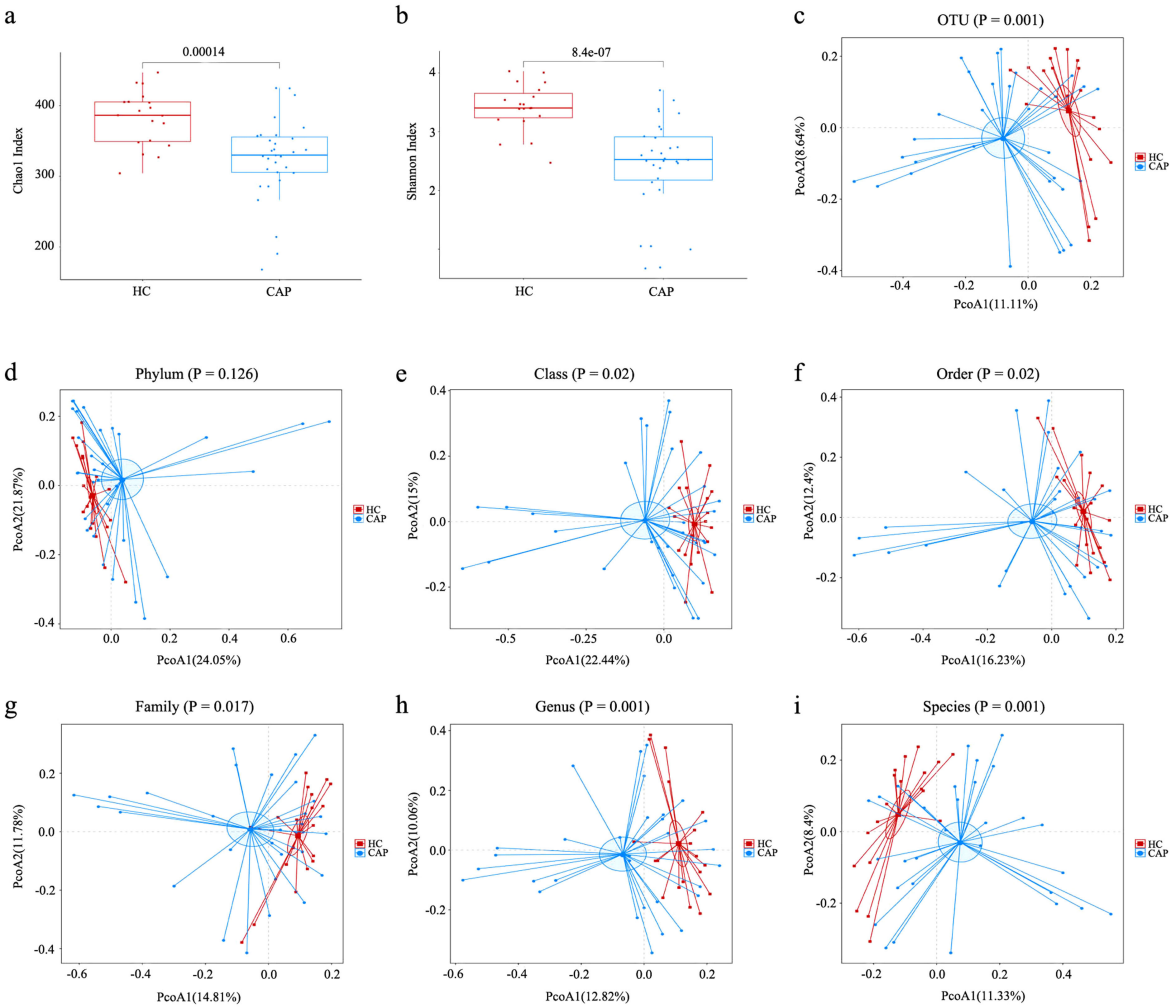
Analysis of alpha- and beta-diversities of gut microbiota between CAP patients and healthy controls. **(a)** Chao1 index; **(b)** Shannon index; Based on the Bray-Curtis algorithm, the principal coordinate analysis (PCoA) of gut microbiota was carried out at the level of OTU **(c)**, phylum **(d)**, class **(e)**, order **(f)**, family **(g)**, genus **(h)** and species **(i)**. n = 31 for the CAP patients and n = 19 for the HC group.

In order to explore the characteristics of gut microbiota in patients with CAP, 16S rRNA sequencing was performed on fecal samples from 31 patients with CAP and 19 healthy controls. In this study, the average sequencing depth of each sample was 60,755 readings, and the number of operational taxonomic units (OTUs) across per single samples ranged from 123 to 370. The Good’s coverage for all samples exceeded 99.5%, indicating that the sequencing depth was sufficient to cover the vast majority of microorganisms in the samples (**Supplementary Table 1**). Moreover, rarefaction curves showed that all sample curves tended to plateau (**Supplementary Figure S1**), which confirms that the volume of sequencing data meets the requirements for microbial diversity analysis. Compared with the HC group, the α-diversity indices (Chao1 and Shannon indices) of gut microbiota in CAP patients were significantly reduced (*p* < 0.001; **Figures 1a, b**), indicating that the microbiota of healthy individuals had higher species richness. Principal coordinate analysis (PCoA) based on the Bray-Curtis algorithm showed that there were significant differences in β-diversity between CAP patients and healthy controls at the level of class, order, family, genus, species, and OTUs (**Figures 1c, e–i**), but no significant difference was observed at the phylum level (**Figure 1d**).

### 3.3 Characteristic analysis of gut microbiota in CAP patients

**Figure 2:**
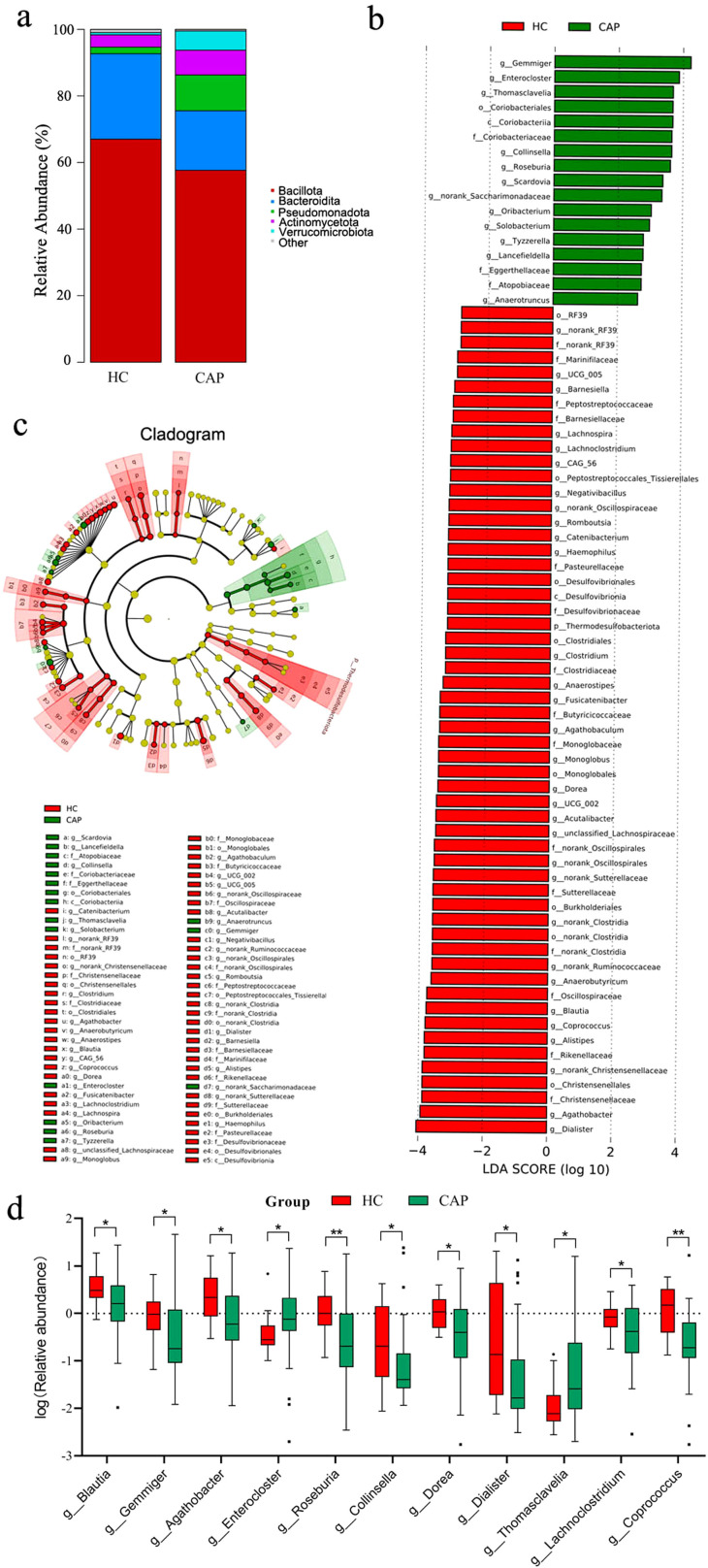
Changes in gut microbiota of CAP patients. **(a)** Relative abundance of gut microbial communities at the phylum level in the HC group and CAP patients; **(b)** LEfSe analysis of differential abundance. The figure shows the linear discriminant analysis (LDA) scores (log10) of major genera: positive scores represent genera with higher abundance in the CAP group, while negative scores represent genera with higher abundance in the HC group; **(c)** Phylogenetic tree. The tree structure reflects the hierarchical distribution of bacteria from phylum to genus, with taxa showing significant differences in abundance marked by colors; **(d)** Displaying the log-transformed relative abundance of genera with significant differences among the top 30 most abundant genera in CAP patients, compared with the healthy control group. *p < 0.05, **p < 0.01. n = 31 for the CAP patients and n = 19 for the HC group.

Analysis of the percentage of relative abundance at the phylum level showed no significant difference between the CAP group and the HC group (**Figure 2a**). At the phylum level, the four most abundant phyla in both groups were Firmicutes, Bacteroidetes, Proteobacteria, and Actinobacteria. The top 10 genera in the CAP group ranked by relative abundance are shown in **Table 3**. Compared with the HC group, there were significant differences among *Blautia, Gemmiger* and *Agathobacter* in the CAP group, but no significant differences were observed in the other genera.

**Table 3:** Top 10 genera with relative abundance in CAP patients.

Genus	CAP group		HC group		*P* value
	Median	IQR	Median	IQR	
*Bacteroides*	11.527	22.058	18.355	12.810	0.194
*Faecalibacterium*	9.208	17.607	11.499	16.073	0.063
*Escherichia - Shigella*	0.899	3.086	0.269	0.441	0.562
*Akkermansia*	0.037	0.038	0.061	0.065	0.082
*Bifidobacterium*	0.391	0.744	1.662	2.938	0.086
*Gemmiger*	0.182	0.931	0.948	1.277	0.032
*Staphylococcus*	0.009	0.010	0.011	0.007	0.466
*Blautia*	1.606	3.041	3.071	3.851	0.029
*Veillonella*	0.018	0.058	0.037	0.051	0.368
*Agathobacter*	0.594	1.997	2.172	4.438	0.020

LEfSe analysis (**Figure 2b**) revealed significant differences of gut microbiota and their abundance between the CAP group and the HC group. The HC group was enriched in genera such as *Dialister, Agathobacter*, and *Alistipes*. In contrast, the CAP group was enriched in genera including *Gemmiger, Enterocloster*, and *Thomasclavelia*. The phylogenetic tree in **Figure 2c** further confirmed these findings, highlighting the significant taxonomic differences in the microbial community structure between the two groups. In addition, among the top 30 genera in the CAP group, 11 genera showed significant differences compared with the HC group (**Figure 2d**), which further supported the LEfSe results and showed different microbial community profiles between the two groups. These findings suggest that the composition and structure of gut microbiota in CAP patients have changed significantly, and there may be characteristic microbial markers associated with the pathological process of CAP, as well as potential regulatory mechanisms underlying this association.

**Figure 3:**
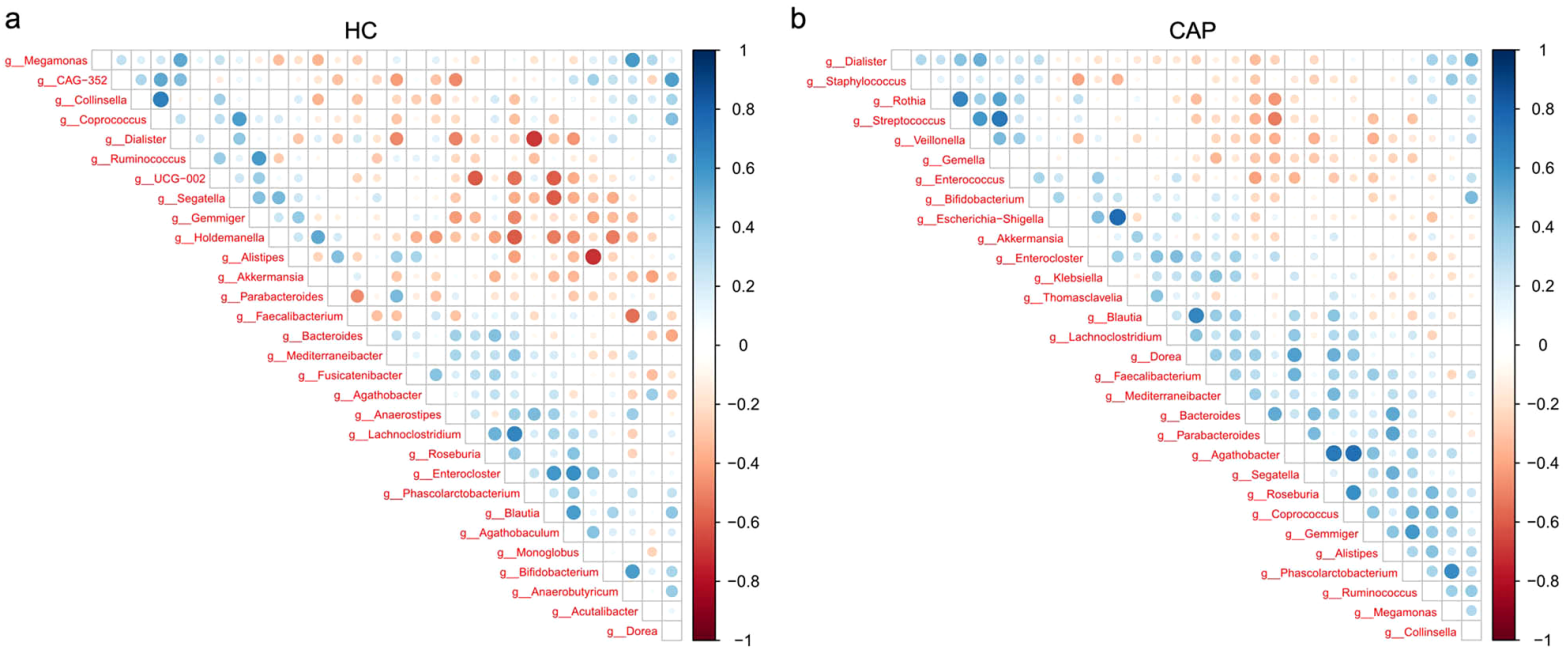
Correlations between gut microbial genera in CAP patients and healthy controls. Correlation heatmap of gut microbial genera in the HC group **(a)** and CAP patients **(b)**. The intensity of the color indicates the strength of the correlation, with blue representing a positive correlation and red representing a negative correlation. n = 31 for the CAP patients and n = 19 for the HC group.

Spearman correlation analysis was performed on the top 30 genera by abundance (**Figure 3**) to explore the interactions of gut microbiota between the HC group and the CAP group. In the HC group, multiple genera showed a significant positive correlation (*p* < 0.05), indicating a stable and synergistic microbial ecosystem (e.g., *Enterocloster, Blautia, Lachnoclostridium* and *Agathobaculum* formed a strong positively correlated cluster). In the CAP group, *Agathobacter* was positively correlated with *Roseburia*, and *Dorea* was positively correlated with *Coprococcus* (*p* < 0.05); in contrast, *Streptococcus* exhibited a significant negative correlation with multiple genera such as *Parabacteroides* and *Bacteroides* (*p* < 0.05).

### 3.4 Predicted functional analysis of gut microbiota in CAP patients

**Figure 4:**
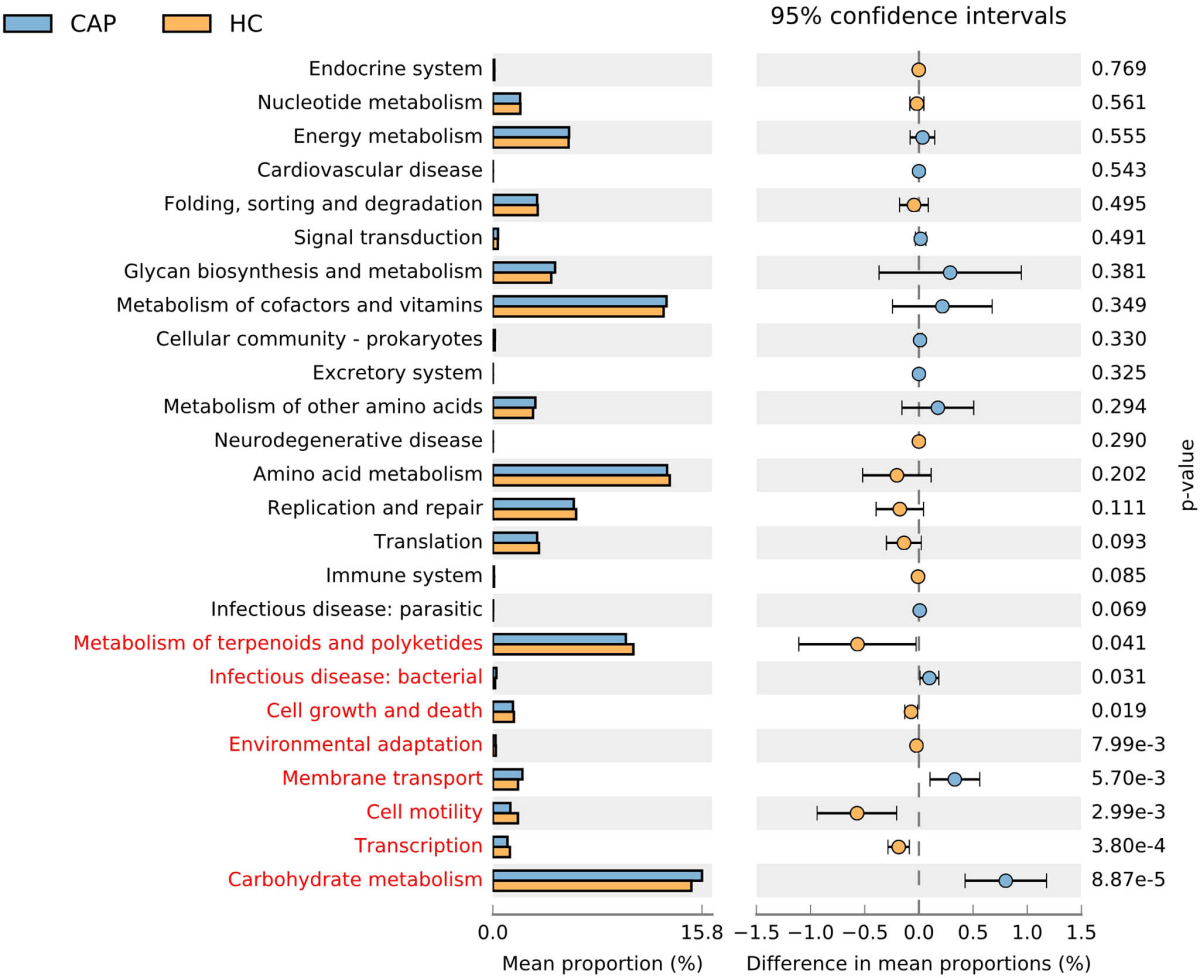
Extended error bar plot and dot plot analysis of PICRUSt-predicted KEGG pathway functions of gut microbiota in CAP patients and healthy controls. Bar plots on the left display the mean proportion of each KEGG pathway, while dot plots on the right show the differences in mean proportions between the two groups of samples using *P*-values. For LEfSe analysis, a logarithmic LDA score threshold > 2.0 and values of *p* < 0.05 were considered statistically significant. n = 31 for the CAP patients and n = 19 for the HC group.

Based on 16S rRNA sequencing data, we used PICRUSt to predict the potential KEGG Ortholog functional profiles of gut microbiota (17). Significant differences were predicted in multiple KEGG functional pathways of microbial communities between CAP patients and healthy controls (**Figure 4**). The microbiota of CAP patients was significantly enriched in functional pathways related to carbohydrate metabolism, membrane transport, and bacterial infection. In contrast, the microbiota of the HC group was significantly enriched in functional pathways associated with metabolism of terpenoids and polyketides, cell growth and death, environmental adaptation, cell motility, and transcription. These findings suggest that there are significant differences in multiple functional characteristics of gut microbiota between the CAP group and the HC group, reflecting the specificity of gut microbiota in metabolic requirements and ecological functions between the two groups. Notably, PICRUSt is a predictive tool based on 16S rRNA sequencing data. Its results are inferential and have certain limitations, so they cannot fully replace direct functional detection methods (e.g., metabolomics).

### 3.5 Analysis of inflammatory response in CAP patients

**Figure 5:**
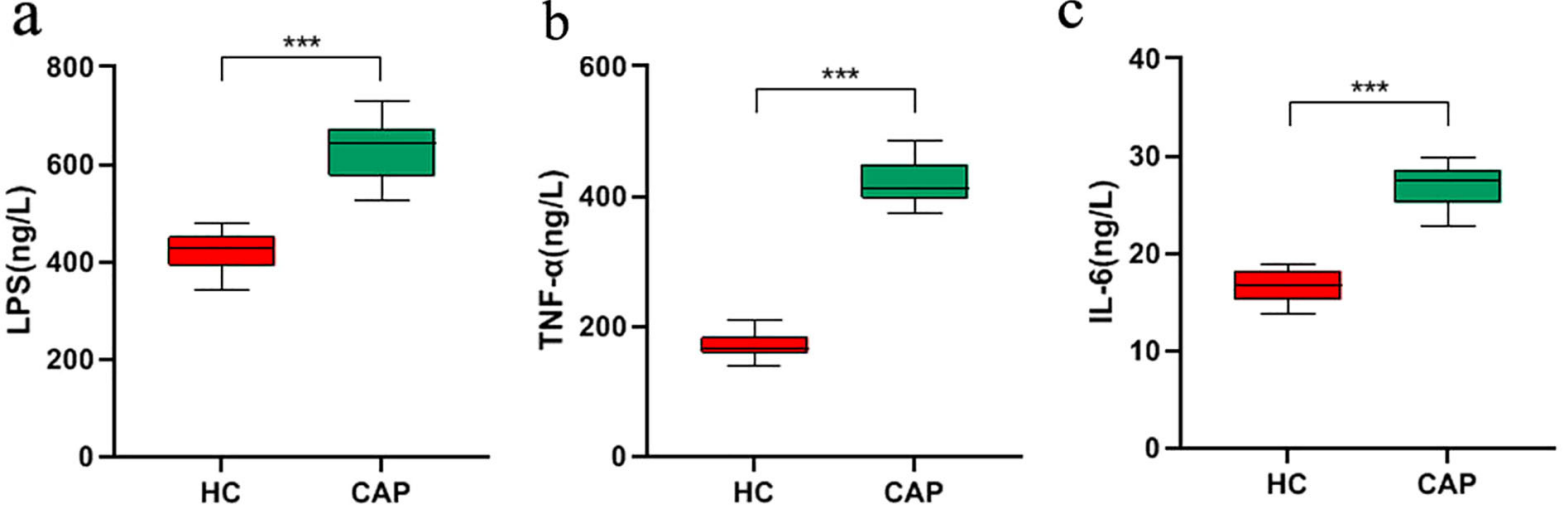
Analysis of inflammation in CAP patients. The expression levels of LPS **(a)**, TNF-α **(b)**, IL-6 **(c)** in the serum of CAP patients and healthy controls were measured by ELISA. ***p < 0.001. n = 31 for the CAP patients and n = 19 for the HC group.

There is a complex bidirectional regulatory relationship between gut microbiota dysbiosis and inflammatory response: microbiota dysbiosis can induce or exacerbate inflammation through multiple mechanisms, while persistent inflammatory status can further disrupt microbiota homeostasis, forming a vicious cycle (18, 19). Therefore, we investigated the expression level of LPS and pro-inflammatory cytokines in CAP patients. We observed that the levels of LPS and proinflammatory cytokines (IL-6 and TNF-α) in the serum of CAP group were significantly higher than those in HC group (*p* < 0.001; **Figure 5**). This result suggests that the elevation of inflammatory indicators in CAP patients may be associated with the microbiota-inflammation vicious cycle, and it also provides indirect evidence for gut microbiota to participate in the regulation of CAP-related inflammation.

## 4 Discussion

In recent years, studies have shown that gut microbiota dysbiosis can induce immune dysregulation and chronic inflammation through the “gut-lung axis,” and it has been proposed that gut microbiota dysbiosis is associated with the pathological mechanism of CAP (8, 20, 21). However, systematic research on the molecular mechanisms of related inflammatory pathways is still lacking. Therefore, this study integrated 16S rRNA gene sequencing and ELISA analysis to explore the role of the gut microenvironment in CAP. In this study, the gut microbiota structure and serum inflammatory markers in CAP patients and healthy controls were systematically analyzed for the first time, which revealed the close relationship between gut microbial dysbiosis and systemic inflammatory response in CAP patients. The results showed that the structure and composition of gut microbiota in CAP patients changed significantly: the levels of serum LPS, TNF-α, IL-6, and hs-CRP increased significantly.

Alpha diversity analysis showed that there were significant differences between the CAP group and the HC group. The Chao1 index, a measure that reflects species richness, and the Shannon index (22), a measure that reflects the overall diversity of samples, were significantly decreased in the CAP group. This indicates that gut microbiota dysbiosis characterized by reduced diversity may be associated with the occurrence of CAP. Beta diversity analysis further supported this conclusion, revealing significant differences in the structure of gut microbiota at the levels of OTU, class, order, family, genus, and species between two groups. These findings suggest that the pathogenesis of CAP may be related to the changes of gut microbiota structure.

Short-chain fatty acids (SCFAs) can enhance the metabolic activity of intestinal epithelial cells, promote mucosal repair, and maintain the integrity of intestinal barrier function (23). In CAP patients, the reduced abundance of *Agathobacter* and *Blautia*, both of which are SCFA-producing bacteria with butyrate as the main metabolic product (24), indicates the assumption that there may be a potential decrease in SCFA synthesis capacity in their intestine. This finding may be consistent with an ‘intestinal leakage’ phenotype. The disruption of epithelial cell junctions makes LPS from intestinal Gram-negative bacteria more likely to enter the bloodstream, resulting in intestinal-derived endotoxemia (25). The elevated serum LPS levels in CAP patients also indirectly confirm the occurrence of this pathological process, suggesting that LPS may contribute to the initiation of systemic inflammation in CAP patients. In the HC group, the enriched genera (e.g., *Dialister* and *Alistipes*) are related to the production of specific SCFAs, and show exhibit anti-inflammatory functions (26, 27). The absence of these genera in CAP patients may be associated with a potential reduction in the intestine’s anti-inflammatory capacity and could contribute to the progression of the vicious cycle between microbiota dysbiosis and inflammation. However, this inference is based on a small sample size, and the differences in genus abundance still require further verification in larger, multi-center cohorts.

In addition, this study found that in CAP patients, the abundances of *Gemmiger, Enterocloster*, and *Thomasclavelia* were significantly higher than those in healthy controls. Studies have reported that *Gemmiger* is enriched in the gut of patients with non-small cell lung cancer (28); *Enterocloster* may be a potential biomarker for patients with irritable bowel syndrome (29); and *Thomasclavelia* is associated with inflammatory biomarkers in patients with inflammatory bowel disease (30). While the role of these genera in CAP remains unclear, based on the above studies, we speculate that they may participate in pro-inflammatory processes through specific pathways, thereby associating with the occurrence and development of CAP. It should be noted that the above results are only preliminary observations, and their specific role in the pathogenesis of CAP still needs to be further verified in larger-scale cohorts.

Results from PICRUSt functional prediction and ELISA detection show that there is a close relationship between the functional characteristics of gut microbiota and inflammation in patients with CAP. Based on this predictive data, we propose the following hypothesis: the enrichment of the predicted functional pathway (carbohydrate metabolism pathway) in the gut microbiota of CAP patients may be associated with the accumulation of acidic products, which in turn could contribute to impaired intestinal barrier integrity, exacerbated intestinal leakage, and increased LPS translocation into the bloodstream (31). Certainly, the above hypothesis is merely a preliminary and hypothesis-generating speculation based on the small-sample results, and its reliability still requires further validation through metabolomics or metagenomics. Meanwhile, the enhancement of predicted functional pathways related to bacterial infection may activate local intestinal inflammation and mediate systemic inflammatory responses through the “gut-lung axis” (5). This speculation is highly consistent with the findings of the present study: the results show that the serum LPS level of CAP patients is significantly increased, and combined with existing studies, it is speculated that this increase may be related to LPS translocation caused by intestinal barrier damage (10, 11). Concurrently, the serum TNF-α and IL-6 levels of patients are significantly elevated, and existing studies have confirmed that LPS can mediate immune cell activation and promote the secretion of these two inflammatory factors (32), providing indirect support for the association between them. In addition, as a key regulatory factor of the acute-phase response, IL-6 may contribute to promoting hepatic synthesis and secretion of CRP (33), which is consistent with the result of increased serum hs-CRP in these patients. In summary, this study preliminarily supports the association between “gut microbiota and systemic inflammation”, provides indirect evidence for the potential role of the “gut-lung axis” in CAP-related inflammation regulation, and also suggests that gut microbiota dysbiosis may play a potential role in the systemic inflammatory response of CAP patients. A large number of recent studies show that probiotics can indirectly reduce inflammatory reaction by restoring intestinal microecological balance; prebiotics can provide nutritional support for beneficial intestinal microorganisms, thereby maintaining their stability and diversity of the gut microbiota; and fecal microbiota transplantation (FMT) has also shown the potential to reshape the balance of intestinal microbial communities and alleviate inflammatory states (34–36). Therefore, interventions based on microbial communities, such as probiotics, prebiotics, or FMT, may become important means to regulate the inflammatory level in patients with CAP.

There are some limitations in this study. First and foremost, this study has a relatively small sample size, which may result in insufficient statistical power and make it difficult to generalize the findings to CAP patients in different regions and across diverse populations. For subsequent studies, it is necessary to expand the sample size and conduct multi-center cohort studies to further verify the association between gut microbiota alterations and CAP. Second, the taxonomic resolution based on 16S rRNA sequencing is limited, which makes it impossible to accurately identify bacteria at the species level, and the concentration of SCFA in serum or feces was not measured in this study. Although the abundance of SCFA-producing bacteria decreased in CAP patients, the effect of this decrease on actual SCFA levels and the direct role of these bacteria in intestinal barrier function have not been confirmed, and further research is needed to clarify. Third, the potential impact of confounding factors on the analysis results concerning the “association between gut microbiota and CAP” cannot be completely ruled out, especially difficult-to-control factors such as diet, long-term use of other types of medications, and comorbidities, which may interfere with the gut microbiota. For future research, it is necessary to optimize the statistical design protocol: through strict control of key confounding factors, the interference of these factors on research results can be minimized, thereby revealing the true association between gut microbiota and CAP more accurately.

## 5 Conclusions

In this study, the gut microbial characteristics and serum inflammatory indicators of CAP patients and healthy controls were systematically compared. Our results suggest a potential association between gut microbiota alterations and systemic inflammation in CAP, and further highlight the potential role of the gut-lung axis in CAP pathogenesis. This work not only fills critical knowledge gaps in the gut-lung cross-talk during CAP but also provides a foundation for future research exploring microbiota-based interventions (e.g., probiotics, prebiotics, or fecal microbiota transplantation) to modulate the gut-lung axis and improve CAP outcomes.

## Data Availability

The data presented in the study are deposited in the Sequence Read Archive (SRA) repository, accession number PRJNA1365037.
