# Supplementary material for: Gut microbiota alterations and systemic inflammation in community-acquired pneumonia: a prospective gut-lung axis study
Source: Front Immunol. 2025 Nov 25;16:1715214. doi: 10.3389/fimmu.2025.1715214 (PMC12685802; doi:10.3389/fimmu.2025.1715214)
Supplement: Supplementary file 1 [file Table1.docx]

Supplementary Material

# Supplementary Figures and Tables

For more information on Supplementary Material and for details on the different file types accepted, please see [here](https://www.frontiersin.org/guidelines/author-guidelines#supplementary-material).

## Supplementary Figures

*
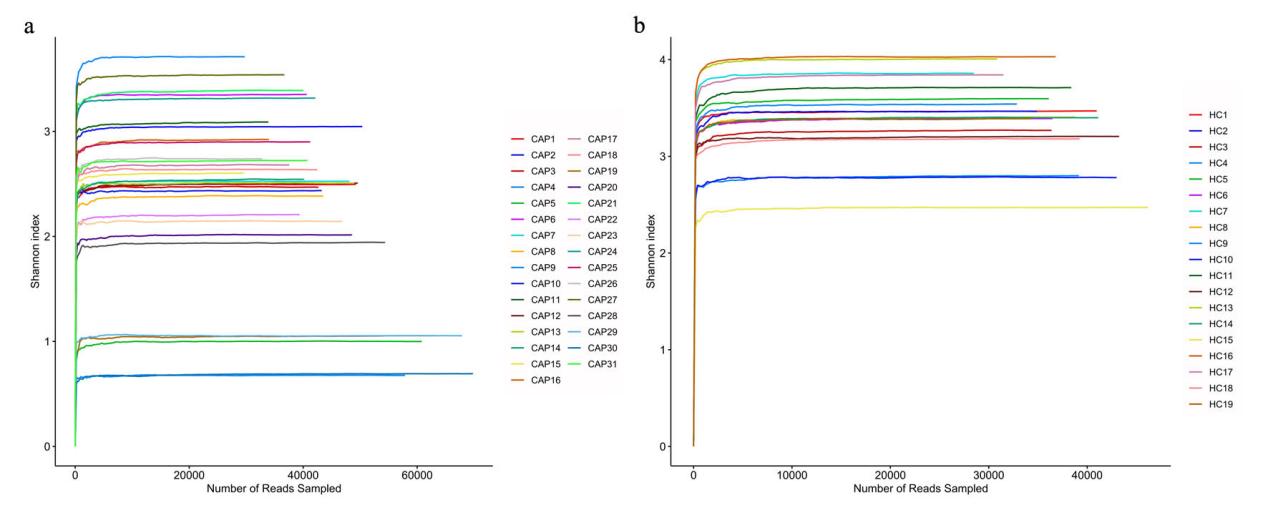
*

**Supplementary Figure 1.** Rarefaction curves in CAP patients (a) and and healthy controls (b).

## Supplementary tables

Supplementary table 1 sequencing depth, OTU numbers, and Good’s coverage in patients and healthy controls

|  | SeqNum | OTUs | Coverage |
| --- | --- | --- | --- |
| CAP1 | 62259 | 260 | 0.9983 |
| CAP2 | 63942 | 177 | 0.9996 |
| CAP3 | 71050 | 262 | 0.9986 |
| CAP4 | 61113 | 123 | 0.9992 |
| CAP5 | 63937 | 219 | 0.9988 |
| CAP6 | 43870 | 259 | 0.9980 |
| CAP7 | 59869 | 273 | 0.9982 |
| CAP8 | 55227 | 289 | 0.9983 |
| CAP9 | 67653 | 327 | 0.9973 |
| CAP10 | 58480 | 282 | 0.9981 |
| CAP11 | 55954 | 246 | 0.9981 |
| CAP12 | 57554 | 240 | 0.9987 |
| CAP13 | 60119 | 263 | 0.9985 |
| CAP14 | 62580 | 280 | 0.9983 |
| CAP15 | 50755 | 190 | 0.9978 |
| CAP16 | 60723 | 283 | 0.9980 |
| CAP17 | 61130 | 282 | 0.9983 |
| CAP18 | 63953 | 254 | 0.9985 |
| CAP19 | 65726 | 230 | 0.9987 |
| CAP20 | 56518 | 246 | 0.9985 |
| CAP21 | 68436 | 318 | 0.9982 |
| CAP22 | 65207 | 252 | 0.9981 |
| CAP23 | 60088 | 217 | 0.9985 |
| CAP24 | 52625 | 309 | 0.9980 |
| CAP25 | 53347 | 240 | 0.9982 |
| CAP26 | 55587 | 258 | 0.9980 |
| CAP27 | 70021 | 323 | 0.9978 |
| CAP28 | 55980 | 219 | 0.9988 |
| CAP29 | 60164 | 219 | 0.9987 |
| CAP30 | 69963 | 146 | 0.9992 |
| CAP31 | 57117 | 248 | 0.9981 |
| HC1 | 63433 | 274 | 0.9980 |
| HC2 | 60386 | 362 | 0.9976 |
| HC3 | 59335 | 280 | 0.9983 |
| HC4 | 51054 | 347 | 0.9978 |
| HC5 | 62603 | 319 | 0.9979 |
| HC6 | 67118 | 343 | 0.9979 |
| HC7 | 80416 | 336 | 0.9970 |
| HC8 | 80847 | 296 | 0.9982 |
| HC9 | 56439 | 316 | 0.9980 |
| HC10 | 57190 | 266 | 0.9986 |
| HC11 | 46850 | 370 | 0.9979 |
| HC12 | 50250 | 287 | 0.9982 |
| HC13 | 58573 | 330 | 0.9979 |
| HC14 | 56725 | 329 | 0.9980 |
| HC15 | 72572 | 278 | 0.9984 |
| HC16 | 61343 | 362 | 0.9981 |
| HC17 | 61822 | 304 | 0.9978 |
| HC18 | 56449 | 256 | 0.9981 |
| HC19 | 63406 | 294 | 0.9982 |
